# Soil Properties Induced Changes in the Microbial Communities Associated With Potato Tubers Grown in Different Lowland Fields of Northern Thailand

**DOI:** 10.1002/mbo3.70092

**Published:** 2025-10-22

**Authors:** Pipat Macharoen, Wuttichai Mhuantong, Thippawan Wannawong, Wiphawee Leesutthiphonchai, Onuma Piasai, Somboon Tanasupawat, Nakarin Suwannarach, Nattakorn Kuncharoen

**Affiliations:** ^1^ Department of Plant Pathology Faculty of Agriculture, Kasetsart University Bangkok Thailand; ^2^ Enzyme Technology Research Team, Biorefinery and Bioproducts Technology Research Group, National Center for Genetic Engineering and Biotechnology National Science and Technology Development Agency Pathum Thani Thailand; ^3^ Department of Biochemistry and Microbiology Faculty of Pharmaceutical Sciences, Chulalongkorn University Bangkok Thailand; ^4^ Center of Excellence in Microbial Diversity and Sustainable Utilization Chiang Mai University Chiang Mai Thailand; ^5^ Department of Biology Faculty of Science, Chiang Mai University Chiang Mai Thailand

**Keywords:** environmental factor, microbial community, microbial function, microbial interaction, potato tuber

## Abstract

Potato tubers are a primary source of infection with phytopathogens, which temporarily colonize their surfaces. Therefore, soil management practices are necessary to reduce pathogen accumulation. In Thailand, potato production typically involves soil amendment and crop rotation to decrease the quantities of soilborne pathogens before and during cultivation. In this study, we investigated the influence of microbial diversity, taxonomy, and functions of bacterial and fungal communities, as well as environmental factors, intra‐ and interkingdom microbial correlations, on potato tubers grown in Tak and Chiang Mai Provinces in northern Thailand, using 16S and ITS amplicon sequencing. The results show that soil properties significantly influenced the species composition of the bacterial and fungal communities on the potato tubers, although they did not distinctly affect overall species richness and evenness. Redundancy analysis also revealed that pH and organic matter are the main factors driving bacterial and fungal taxon enrichment and reduction. These factors also affect microbial composition and enhance the stability of cooperative and competitive microbial interactions. These findings demonstrate that pH and organic matter potentially impact the fluctuation of beneficial and phytopathogenic bacterial and fungal quantities in the potato tuber microbiome. Therefore, a comprehensive understanding of these dynamics could help us develop environmentally friendly strategies for supporting farming practices by promoting beneficial microbial interactions.

## Graphical abstract

Microbial communities on potato tubers in northern Thailand were studied with 16S and ITS amplicon sequencing. It was found that soil pH and organic matter are key drivers of bacterial and fungal composition, affecting the balance between pathogens and beneficial microbes. This discovery suggests that modifying soil properties can be a strategy for boosting crop production and sustainable disease control.

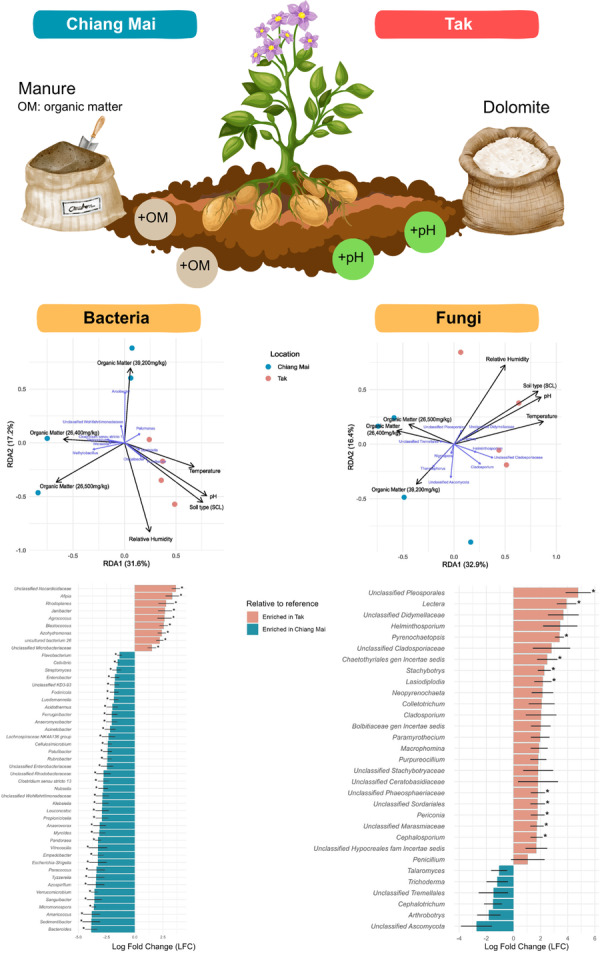

**Abbreviations**
AMPK: Adenosine monophosphate‐activated protein kinase
CL: Clay loam
DADA2: Divisive Amplicon Denoising Algorithm version 2
ITS: Internal transcribed spacer
MAPK: Mitogen‐activated protein kinase
PICRUSt2: Phylogenetic investigation of communities by reconstruction of unobserved states version 2
SCL: Sandy clay loam

## 1 Background

The potato (*Solanum tuberosum* L.) is a hidden nutritional treasure and a hunger‐combatting carbohydrate‐rich crop (Lutaladio and Castaldi 2009). It is the world's fourth‐largest crop after rice, wheat, and maize, and the leading non‐grain food crop, with a global annual yield of approximately 376 million tons (Singh and Sandhu 2023; FAOSTAT 2024). Potatoes are among the most essential vegetable crops for food and feed production. They are extraordinarily significant for ensuring global food security and famine eradication, as no other crop can match the potato's production per unit area, and its price remains relatively stable (Devaux et al. 2014; Amare et al. 2022). Moreover, fresh potato consumption currently accounts for over two‐thirds of the harvest, and over 1.3 billion people worldwide consume potatoes as a staple food (~50 kg/person/year). As a result, the global potato demand has doubled since 1993 (Devaux et al. 2021). Potato production in Thailand has increased significantly in recent years, driven by a rising demand for both fresh and processed potatoes. Most potatoes are produced in Northern Thailand in a single winter crop (November–March) following rice or corn cultivation in lowland areas (300–700 m above sea level), primarily in Chiang Mai and Tak Provinces (Kittipadakul et al. 2016).

The potato tuber is an excellent source of nutrients and vitamins, enabling it to enhance diets and reduce mortality rates related to malnutrition (Lutaladio and Castaldi 2009). It forms the below‐ground part of the shoot, which is anatomically distinct from the roots (Fernie and Willmitzer 2001). Tubers serve as the primary source of infection for many potato plant pathogens, which temporarily colonize the tubers' surfaces. As infected tubers often remain symptomless, seed tubers can spread pathogens over long distances (van der Wolf and De Boer 2007). The potato tuber is colonized by a diverse microbial consortium during growth (Weinert et al. 2010); however, this has scarcely been investigated. Previous studies on potato‐tuber‐associated microbes have focused on bacterial and fungal pathogens that cause potato tuber diseases, such as *Streptomyces scabiei* (Henao et al. 2022), *Pectobacterium* spp. (Han et al. 2023), *Dickeya* spp. (van der Wolf et al. 2021), *Fusarium oxysporum* (García Bayona et al. 2011), *Rhizoctonia solani* (Yanar et al. 2005), *Phytophthora infestans* (Rani et al. 2023), and endophytic microbes like *Bacillus subtilis*, *Bacillus mojavensis*, and *Klebsiella variicola* (Shirazi et al. 2023). Only Weinert et al. (2010) have studied bacterial diversity on potato tuber surfaces by isolating and identifying microbes using a 16S rRNA gene sequence. They reported that genera from *Bacillus*, *Lysobacter*, *Dickeya*, *Streptomyces*, *Flavobacterium*, and *Ensifer* were predominant on the tubers' surfaces. Meanwhile, the fungal diversity on potato tubers in fields has only been investigated by Zimudzi et al. (2018), who used next‐generation sequencing via the Illumina MiSeq platform. They revealed that the predominant genera found on the potato tubers were *Peyronellaea*, *Fusarium*, *Thielavia*, *Setophoma*, and *Chaetomium*.

Along with soil physicochemical properties and plant exudates, the chemicals used for soil amendments have recently been shown to constitute one of the key factors shaping the associated microbial community in the rhizosphere and geocaulosphere—the soil in contact with potato tuber surfaces (Yu et al. 2023). In Thailand, the Atlantic potato is the most popular cultivar, grown in approximately 90% of the total cultivation area, because it requires a shorter growing season (90‒100 days) and serves as the primary raw material for the processing of the potatoes into chips and snack foods (Kittipadakul et al. 2016). There are two main practices used for potato production in the lowland fields of Northern Thailand. One is growing potatoes after paddy rice with manure, as flooding helps destroy soilborne phytopathogens; this practice is commonly used in Chiang Mai Province. The other approach is to apply lime (CaO) or dolomite [CaMg(CO_3_)_2_] to the soil to alter its microbial biomass before cultivating the potatoes, which is typically employed in Tak Province (Kittipadakul et al. 2016; Longwe et al. 2023).

Historically, research into microbial communities has provided a limited view of their diversity, as it predominantly focused on cultivable bacteria, highly abundant taxa, or microorganisms categorized solely by their morphological or phenotypic characteristics (Manzoni et al. 2012). Recently, advances in next‐generation sequencing methodologies, such as amplicon sequencing, have represented a paradigm shift in characterizing microbial diversity. These tools facilitate the analysis of community composition at an unprecedentedly high level of phylogenetic resolution. Amplicon sequencing, also referred to as metabarcoding, is a polymerase chain reaction (PCR)‐based technique that specifically amplifies variable DNA regions within evolutionarily conserved phylogenetic or functional marker genes. It also represents the most cost‐effective and rapid method for studying microbial communities in various environments, including those containing unculturable microorganisms (Alteio et al. 2021). Marker genes commonly used to study microbial communities include the 16S rRNA gene for bacteria and archaea, as well as the internal transcribed spacer (ITS) regions for fungi. These markers are favored due to their high accuracy in taxonomic resolution and the availability of numerous curated databases (Hrovat et al. 2024; Edwards et al. 2019).

Different field sites have different soil microbial communities, which are the primary sources of microbes colonizing belowground plant compartments (Buchholz et al. 2019), raising the question of how treating soil with manure and dolomite influences the potato tuber microbiota in terms of microbial diversity, taxonomic composition, function, and microbial correlation. To address this question, we studied the effect of soils that were either treated or untreated on the microbial communities of potato tubers grown in natural fields in Tak and Chiang Mai Provinces, Thailand. Our goal was to unravel the microbial diversity, taxonomic composition, and predicted functions of the microbiomes of potato tubers grown on land subjected to manure and dolomite amendments using 16S and ITS amplicon sequencing, which are highly informative gene markers for identifying bacteria and fungi, respectively. We also compared the interkingdom microbial interactions of potato tubers grown in fields to gain insights into the stability of the communities and the microbes that tend to co‐occur. Our work provides foundational data for a better understanding of the effect of environmental factors on potato‐tuber‐associated microbiota and offers valuable guidelines for adopting appropriate potato production practices that promote high yields and reduce diseases.

## 2 Materials and Methods

### 2.1 Sampling Sites and Sample Collection

The fully developed potato tubers (from the Atlantic cultivar) were collected at the senescent stage, 85–90 days after planting, according to the method reported by Hack et al. (1993), from eight natural fields: two in the subdistrict of Long Khot and two in the subdistrict of Mae Ho Phra, Chiang Mai Province, Thailand, on February 10, 2023, and four in Ruam Thai Pattana, Phop Phra District, Tak Province, on March 18, 2023 (Table 1). All the fields sampled had a potato‐planting history of over 20 years. Before they were sampled, each field was split into four plots (presenting a W pattern) to ensure the crops were minimally disrupted. In each plot, 20 tubers of equal size (four tubers per sampled plant) with tightly adhering soil were collected, amounting to 80 tubers per field. All samples from each field were kept in sterile polyethylene bags, stored at low temperature in an ice box (~4°C), and transported to our laboratory within 12 h. Coarse soil particles were removed from the collected samples. Then, the tubers' outer peel layers (ectodermis) with tightly adhering soil particles were carefully peeled off with a sterile knife. Thus, the tuber surfaces not only included microbial communities colonizing the soil attached to these surfaces but also those colonizing the potato tuber ectodermis. Ten grams of the potato peel layers were transferred into a sterile mortar and pestled with liquid nitrogen to obtain the peel layer powder. Each sampled powder was kept at −20°C for further processing. Bulk soil samples were also randomly sampled to analyze basic physicochemical properties. Sample data, cultural practices, location, and meteorological parameters (temperature and humidity) were recorded.

**Table 1:** Potato tuber sampling sites.

Field name	Geographic coordinate	Subdistrict	District	Province	Relative humidity	Temperature	Yield (kg/hectare)
TLKCD‐F1	19°06′06.3″ N 99°10′51.2″ E	Long Khot	Phrao	Chiang Mai	58.9	23.3	18,262.5
TLKCD‐F2	19°06′06.3″ N 99°10′51.2″ E	Long Khot	Phrao	Chiang Mai	59.1	23.1	18,262.5
TMTTD‐F1	19°06'46.8″ N 99°01′04.2″ E	Mae Ho Phra	Mae Taeng	Chiang Mai	51.5	23.4	18,262.5
TMTTD‐F2	19°06'46.8″ N 99°01′04.2″ E	Mae Ho Phra	Mae Taeng	Chiang Mai	52.1	23.6	18,262.5
TPPTD‐F1	16°30′14″ N 98°46′51″ E	Ruam Thai Pattana	Phop Phra	Tak	62.1	24.1	17,312.5
TPPTD‐F2	16°30′14″ N 98°46′51″ E	Ruam Thai Pattana	Phop Phra	Tak	61.9	24.2	17,312.5
TPPTH‐F1	16°26′2″ N 98°46′17″ E	Ruam Thai Pattana	Phop Phra	Tak	63.2	25.5	17,312.5
TPPTH‐F2	16°26′2″ N, 98°46′17″ E	Ruam Thai Pattana	Phop Phra	Tak	63.5	25.3	17,312.5

### 2.2 Analysis of Soil Physicochemical Properties

The basic physicochemical properties of the soil samples from each field were determined, including pH in water using a pH meter equipped with glass and reference electrodes (Thomas 1996); soil classification by particle size using a hydrometer (Gee and Bauder 1979); soil organic carbon (SOC) via oxidizing carbon with acidic dichromate, followed by applying the method developed by Walldey and Black (1934); available phosphorus using the Bray and Kurtz P‐1 method (Bray and Kurtz 1945); available potassium using the Ammonium Acetate method (Helmke and Sparks 1996); and available calcium and magnesium using atomic absorption spectrophotometry (Suarez 1996) at the Department of Soil Science, Faculty of Agriculture, Kasetsart University, Bangkok, Thailand. The results regarding soil physicochemical properties are shown in Table 2.

**Table 2:** Soil physicochemical properties.

Field name	pH	Soil type	Organic matter (mg/kg)	Available phosphorus (mg/kg)	Available potassium (mg/kg)	Available calcium (mg/kg)	Available magnesium (mg/kg)
TLKCD‐F1	4.6	CL	26,400	80.8	345	1265	201
TLKCD‐F2	4.7	CL	26,500	81.8	350	1197	212
TMTTD‐F1	4.9	CL	39,200	50.8	322	1845	365
TMTTD‐F2	4.8	CL	39,300	52.8	323	1865	377
TPPTD‐F1	6.6	SCL	15,700	91.5	540	1650	443
TPPTD‐F2	6.7	SCL	15,900	90	533	1590	439
TPPTH‐F1	6.8	SCL	15,600	91.8	540	1600	440
TPPTH‐F2	6.7	SCL	15,900	93	545	1650	443

Abbreviations: CL, clay loam; SCL, sandy clay loam.

### 2.3 DNA Extraction and Amplicon Sequencing

Each potato peel layer powder sample was weighed (0.2 g) and transferred into the PowerBead Tube of a DNeasy PowerSoil Pro Kit (Qiagen, Hilden, Germany), and DNA was extracted according to the manufacturer's instructions. The purity and concentration of the extracted DNA of each sample were determined at an absorbance ratio of 260/280 (1.8–2.0) using a NanoDrop Eight Spectrophotometer (Thermo Fisher Scientific, Wilmington, DE, USA).

Each sampled DNA was used for library construction and amplicon sequencing. For the bacterial community, the V5–V7 hypervariable region of the 16S rRNA gene was amplified using the primer set of 799F (5′‐AACMGGATTAGATACCCKG‐3′) and 1193R (5′‐ACGTCATCCCCACCTTCC‐3′), which showed low affinity for nontarget DNA such as chloroplast and mitochondrial DNA (Beckers et al. 2016). For the fungal community, the ITS2 was amplified using the universal primer sets ITS3‐2024F (5′‐GCATCGATGAAGAACGCAGC‐3′) and ITS4‐2409R (5′‐TCCTCCGCTTATTGATATGC‐3′) for fungus (White et al. 1990). All amplification conditions consisted of initial denaturation at 98°C for 1 min, followed by 30 cycles of denaturation at 98°C for 10 s, annealing at 50°C for 30 s, and elongation at 72°C for 30 s. Finally, 72°C was applied for 5 min (Novogene Tianjin, China). The library was sequenced on an Illumina NovaSeq 6000 platform, generating 250 bp paired‐end reads (Illumina, San Diego, USA).

### 2.4 Bioinformatics and Data Analysis

The raw Illumina paired‐end sequences were initially assessed for quality using FASTQC (Andrews 2010) and MultiQC (Ewels et al. 2016). Sequences with an average Phred score below 30 were excluded, and reads were trimmed with respect to barcode and primer sequences using FASTP (Chen et al. 2018). Paired‐end reads were subsequently merged with FLASH (Magoč and Salzberg 2011). The merged and quality‐filtered sequences of the microbial communities were analyzed based on the workflow in QIIME 2 version 2022.2 (Bolyen et al. 2019).

For 16S rRNA gene sequences, reads with lengths over 400 bp were denoised, chimeras were removed, and the reads were clustered into amplicon sequence variants (ASVs) using DADA2 (Callahan et al. 2016) as implemented in QIIME2. The parameters were set with a minimum total frequency of 10 and a requirement that each ASV be observed in at least two samples. Representative sequences for each ASV were taxonomically classified using a BLAST+ consensus taxonomy classifier against the SILVA database (Quast et al. 2013) (version 138.1) with an 80% identity cutoff. Sequences classified as chloroplast or mitochondria were removed before further analysis. For the ITS2 region, sequences with lengths over 295 bp were selected and processed with DADA2 to construct ASVs. Taxonomic classification of the fungal community was performed using a consensus BLAST approach against the UNITE database (Abarenkov et al. 2024).

Redundancy Analysis was performed in the vegan package in R version 4.3.2 (Oksanen et al. 2013), and microbial diversities were estimated through the alpha diversity index, including species richness, the Chao1 richness estimator, and the Shannon diversity index from the ASV table. Beta diversity analysis based on the Bray–Curtis dissimilarity matrix was used to measure the similarity across the samples. The significant effects of dolomite amendment in terms of shaping microbial communities were calculated via a permutational multivariate analysis of variance (PERMANOVA) using 999 permutations. Functional gene predictions based on the 16S rRNA and ITS2 amplicon sequencing data were performed using PICRUSt2 (Douglas et al. 2020) and FunFun (Krivonos et al. 2023), respectively.

### 2.5 Differential Abundance and Correlation Analysis of Microbial Communities

Differences in microbial composition were statistically calculated using the analysis of compositions of microbiomes with bias correction (ANCOM‐BC) method (Lin and Peddada 2020). Significant differences in the predicted pathway composition were assessed using White's nonparametric *t*‐test in STAMP (Parks et al. 2014). Correlation of environmental factors was calculated using the Hmisc package in R version 4.3.2 (Harrell 2019), and intra‐ and interkingdom correlations were computed using Pearson correlation analysis via the SciPy package in Python (Virtanen et al. 2020).

## 3 Results

### 3.1 Sequencing Data Summary

The total reads of all the samples amounted to 2,856,030 for 16S rRNA and 1,171,056 for ITS. After filtering and removing low‐quality, mitochondrial, chloroplast, and chimeric sequences, the remaining reads amounted to 712,068 for 16S rRNA (mean: 89,008 reads) and 125,244 for ITS (mean: 15,655 reads). The bacterial and fungal reads were clustered into ASVs, ranging from 581 to 1233 and 37 to 68 ASVs, with mean lengths of 369 and 295 bp, respectively.

### 3.2 Taxonomic Composition of Microbial Community Structure

**Figure 1:**
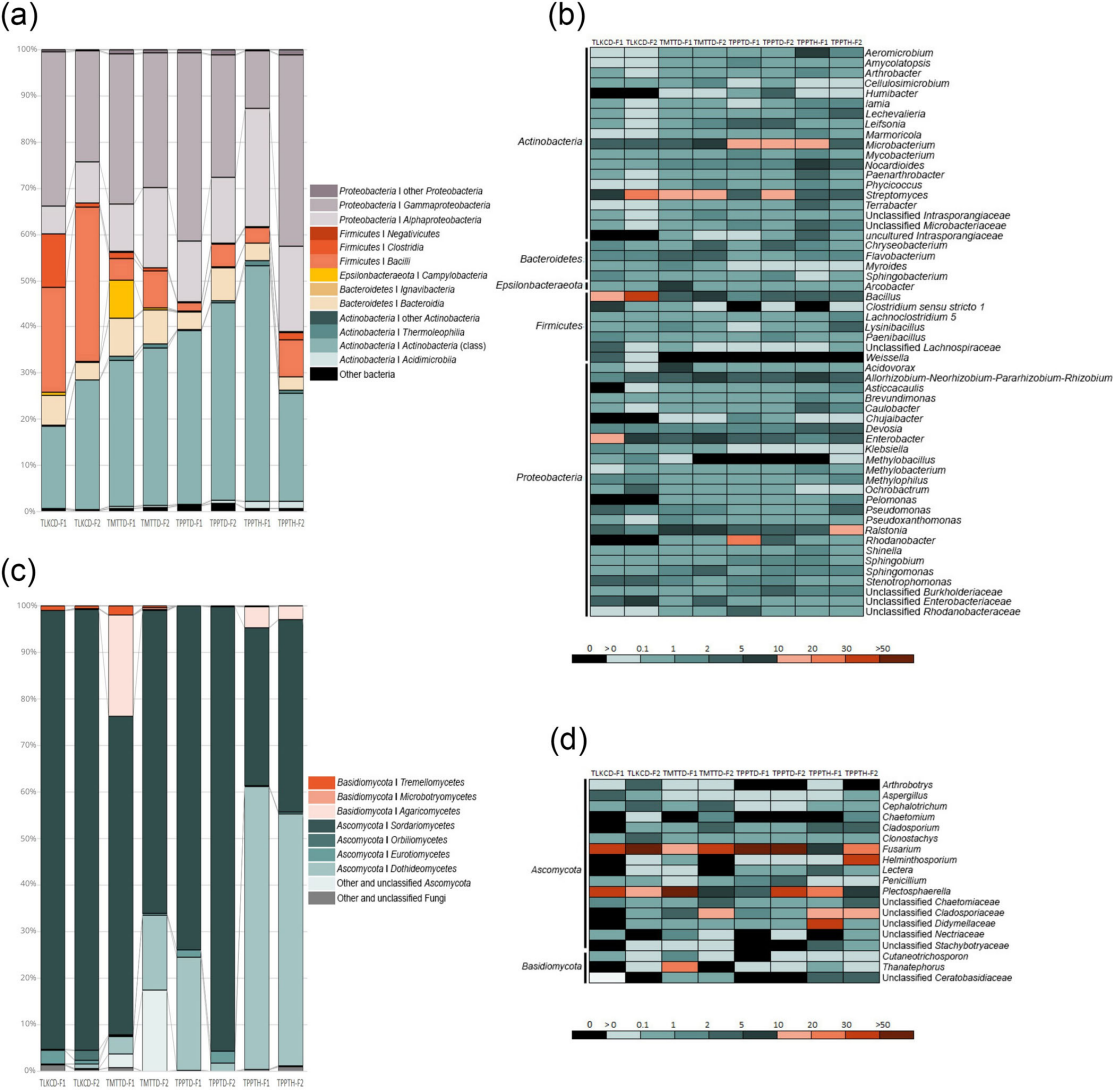
The relative abundances of potato‐tuber‐associated bacteria (a) at the phylum and class levels and (b) the genus level and potato‐tuber‐associated fungi (c) at the phylum and class levels and (d) the genus level under two different fields (*n* = 4). TLKCD‐F1, TLKCD‐F2, TMTTD‐F1, and TMTTD‐F2 are fields form Chiang Mai, while TPPTD‐F1, TPPTD‐F2, TPPTH‐F1, and TPPTH‐F2 are fields form Tak.

The most abundant bacterial phyla associated with the potato tubers were *Proteobacteria* (33.1%–61.1%) and *Actinobacteria* (18.3%–53.8%), followed by *Firmicutes* (2.0%–34.4%), *Bacteroidetes* (2.8%–8.3%), and *Epsilonbacteraeota* (0.1%–8.2%). Other phyla with an abundance of less than 1%, including *Acidobacteria*, *Verrucomicrobia*, *Gemmatimonadetes*, and *Armatimonadetes*, were found as minor components (Figure 1a). *Actinobacteria* and *Gammaproteobacteria* were the most abundant bacterial classes in all the samples, followed by *Alphaproteobacteria* and *Bacilli*. The top 10 bacterial genera in all the potato tuber microbial communities were *Streptomyces*, *Bacillus*, *Microbacterium*, *Ralstonia*, *Enterobacter*, *Allorhizobium*‐*Neorhizobium*‐*Pararhizobium*‐*Rhizobium*, *Rhodanobacter*, *Nocardioides*, *Devosia*, and *Pseudomonas* (Figure 1b).

The fungal communities predominantly comprised the phylum *Ascomycota*, accounting for almost 80% of the reads in all the sampled potato tubers, followed by *Basidiomycota*, which constituted a smaller proportion and was present in only some of the samples (Figure 1c). The genera *Chaetomium* and *Arthrobotrys* were less abundant in most of the potato tubers grown in dolomite‐amended soil, while the genus *Cephalotrichum* was slightly more abundant in the potato tubers cultivated in Chiang Mai (Figure 1d). Furthermore, some taxa were more abundant in particular sample groups. For example, unclassified *Didymellaceae* members were more dominant in the potato tubers grown in Tak (TPPTH‐F1), and unclassified *Cladosporiaceae* members were more abundant in the potato tubers cultivated in Tak that were (TPPTH‐F1 and TPPTH‐F2) and were not (TLKCD‐F1) subjected in Chiang Mai (Figure 1d).

**Figure 2:**
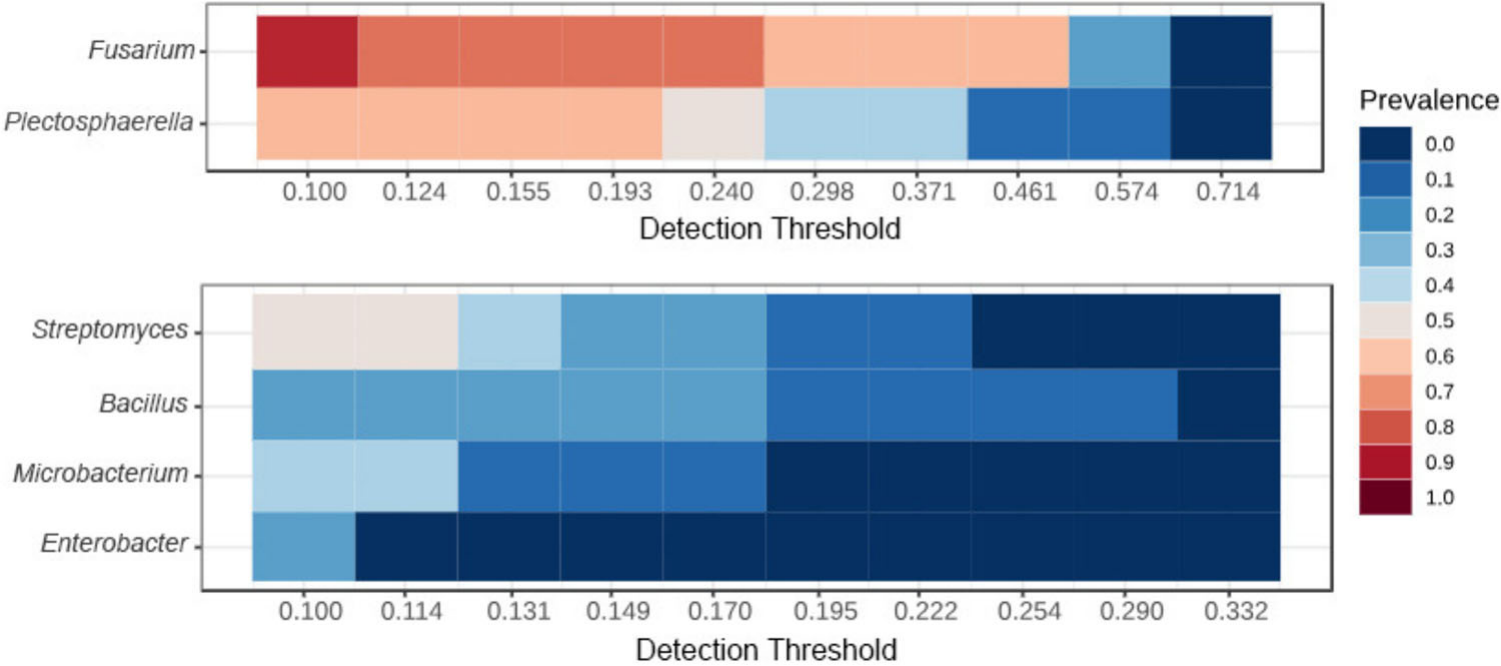
The core microbial taxa associated with potato tubers grown in Chiang Mai and Tak.

Analysis of the core microbiome (Figure 2) revealed that *Streptomyces*, *Bacillus*, *Microbacterium*, and *Enterobacter* represented the core bacterial genera of the potato tubers, whereas *Fusarium* and *Plectosphaerella* were the core fungal genera and the most abundant genera.

### 3.3 Influence of Environmental Factors on Bacterial and Fungal Communities Across Regions

**Figure 3:**
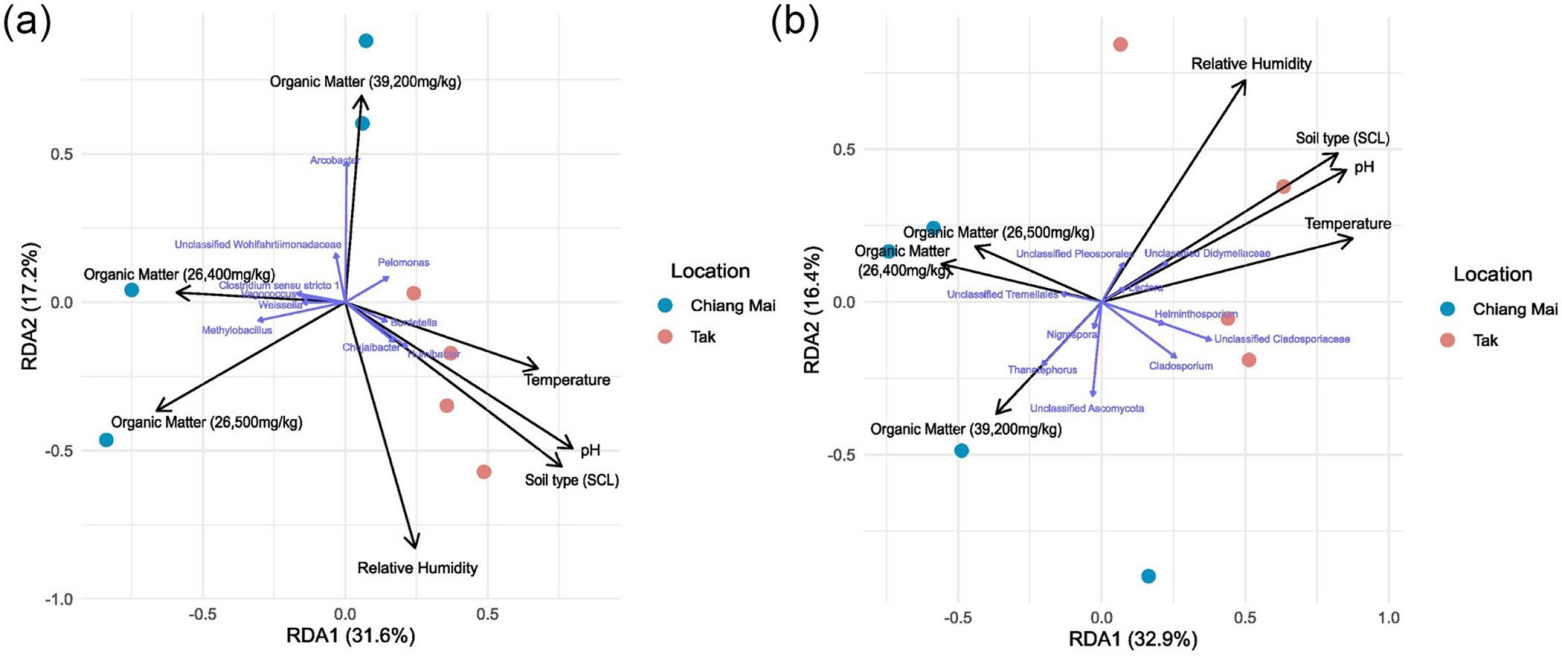
The Redundancy analysis of (a) bacterial and (b) fungal communities associated with potato tubers grown in Chiang Mai and Tak.

Environmental factors significantly shape the bacterial and fungal communities in samples from Chiang Mai and Tak. Variables such as temperature, pH, soil type, relative humidity, and varying levels of organic matter significantly influence both communities. Tak samples cluster toward higher temperatures, soil types, and pH levels, while Chiang Mai samples align more closely with higher organic matter. In the bacterial community, taxa such as *Methylobacillus*, *Vagococcus*, *Weissella*, and *Acrobacter* are closely linked to Chiang Mai condition (Figure 3a). In contrast, *Pelomonas*, *Bordetella*, *Chujaibacter*, and *Humibacter* are more associated with Tak condition. For the fungal community, *Nigrospora* and *Thanatephorus* are associated with Chiang Mai, while *Lectera*, *Helminthosporium*, and *Cladosporium* show stronger associations with environmental conditions in Tak (Figure 3b). These findings indicate that environmental factors, such as pH, organic matter, and temperature, strongly influence the species composition and relative abundance within the communities.

### 3.4 Microbial Diversity of Potato Tubers

**Figure 4:**
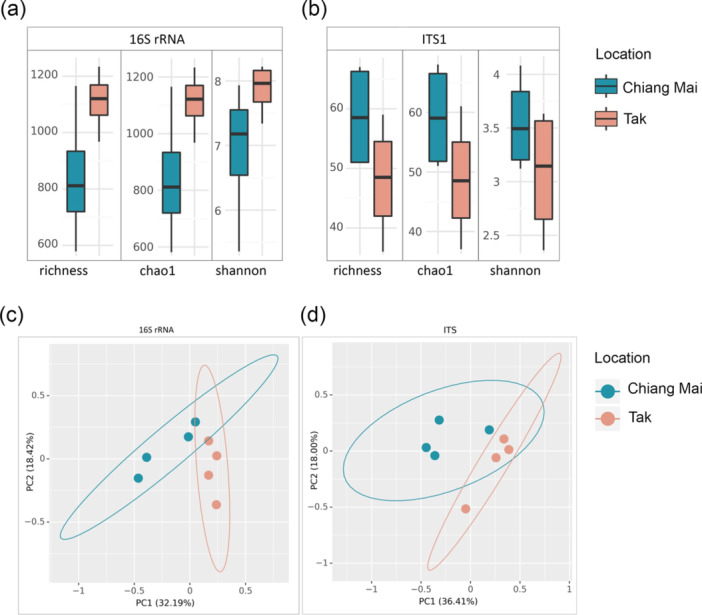
The alpha diversity of (a) bacterial and (b) fungal communities associated with potato tubers grown in Chiang Mai and Tak, determined according to the richness, Chao1, and Shannon indices, respectively (*n* = 4). The PCoA depicts the beta diversity of (c) bacterial and (d) fungal community compositions (*n* = 4) based on the Bray–Curtis distance.

Based on microbial diversity analyses, the tubers grown in Tak had greater bacterial richness and higher values of alpha diversity indices (Chao1 and Shannon) (Figure 4a). Meanwhile, the potato tubers cultivated in Chiang Mai had greater fungal richness and higher values of diversity indices (Chao1 and Shannon) (Figure 4b). However, these differences were not statistically significant (*p* > 0.05). Conversely, beta diversity analysis showed that the structures of the fungal and bacterial communities associated with potato tubers significantly changed (*p* < 0.05), indicating differences in community composition between the sample groups. The principal coordinate analysis (PCoA) plots clearly illustrate the separation of microbial communities under different potato growth conditions in Chiang Mai and Tak (Figure 4c,d).

### 3.5 Differences in Potato Tuber Microbial Community Composition Resulting From Different Environmental Conditions

**Figure 5:**
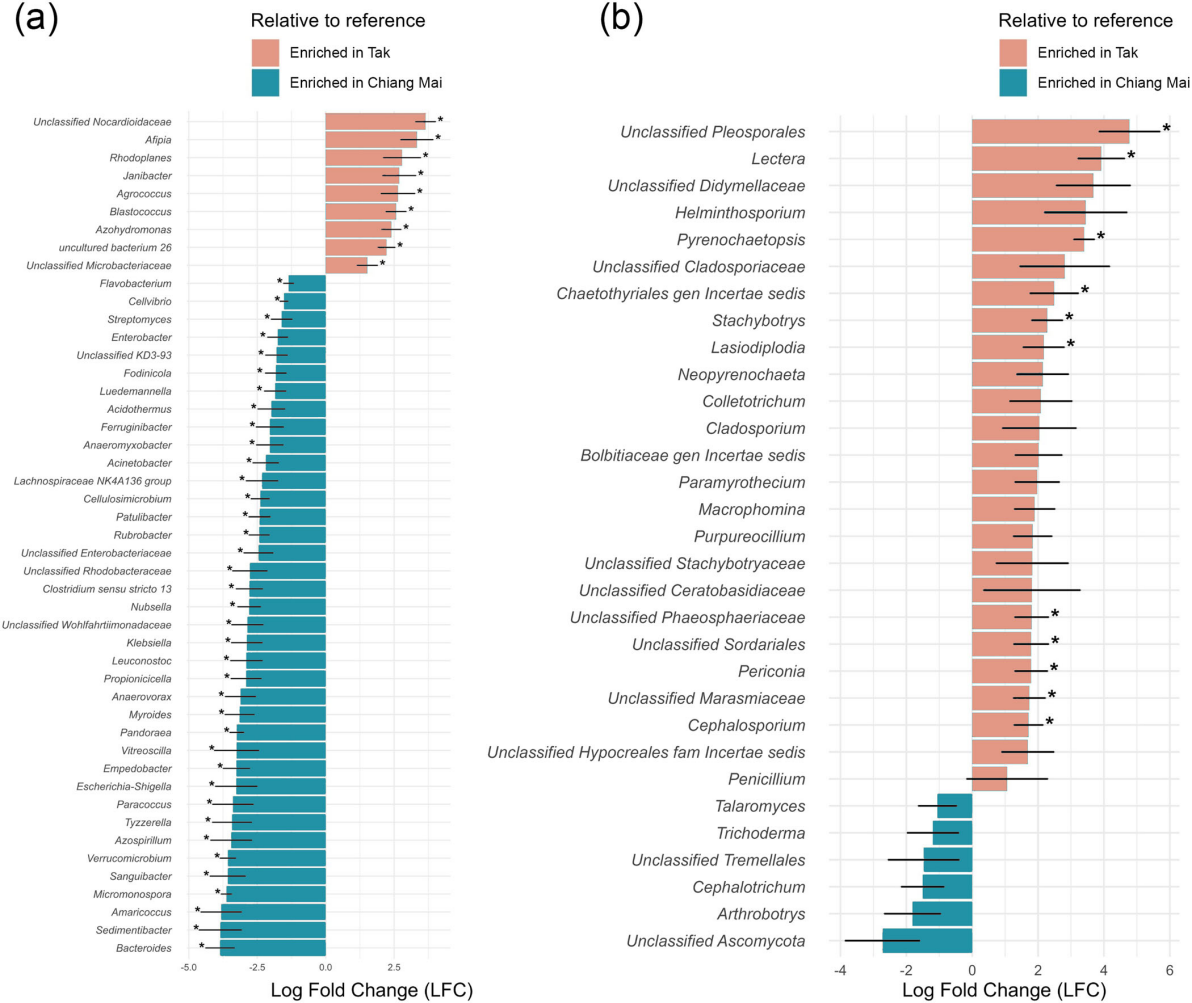
Differential abundant (a) bacterial and (b) fungal genera associated with potato tubers based on the ANCOM‐BC analysis. Pairwise differential abundance analysis of bacterial genera associated with potato tubers grown in Chiang Mai compared to Tak. Genera that differentiate significantly between the groups are displayed when adjusting for sibling effects, with effect size (additive log ratio) and 95% confidence error bars. All effect sizes with Holm's correction *p*‐values < 0.05 are displayed.

Differential abundance analysis of the microbiome demonstrated that Tak province significantly (*p* < 0.05) increased the abundance of bacterial genera (Figure 5a) such as *Afipia*, *Rhodoplanes*, *Janibacter*, *Agrococcus*, *Blastococcus*, and *Azohydromonas*. Conversely, it decreased the abundance of *Bacteroides*, *Sedimentibacter*, *Amaricoccus*, *Micromonospora*, *Sanguibacter*, *Verrucomicrobium*, *Azospirillum*, *Tyzzerella*, *Paracoccus*, *Escherichia*‐*Shigella*, *Empedobacter*, *Vitreoscilla*, *Pandoraea*, *Myroides*, *Anaerovorax*, *Propionicicella*, *Leuconostoc*, *Klebsiella*, *Nubsella*, *Clostridium sensu stricto 13*, *Rubrobacter*, *Patulibacter*, *Cellulosimicrobium*, *Lachnospiraceae NK4A136 group*, *Acinetobacter*, *Anaeromyxobacter*, and *Ferruginibacter*, which are enriched in Chiang Mai province. In the fungal communities (Figure 5b), *Lectera*, *Pyrenochaetopsis*, *Stachybotrys*, *Lasiodiplodia*, *Periconi*, and *Cephalosporium* showed a significant (*p* < 0.05) increase in abundance in the potato tubers grown in Tak province. However, the abundance of *Arthrobotry*, *Cephalotrichum*, *Trichoderma*, *Talaromyces*, *Nigrospora*, and *Fusicolla* was reduced, although the difference was not significant (*p* > 0.05), indicating that Tak province was able to increase the abundance of fungi but not in Chiang Mai province.

### 3.6 Correlations Pertaining to the Microbial Compositions of Potato Tubers Grown on Tak and Chiang Mai

**Figure 6:**
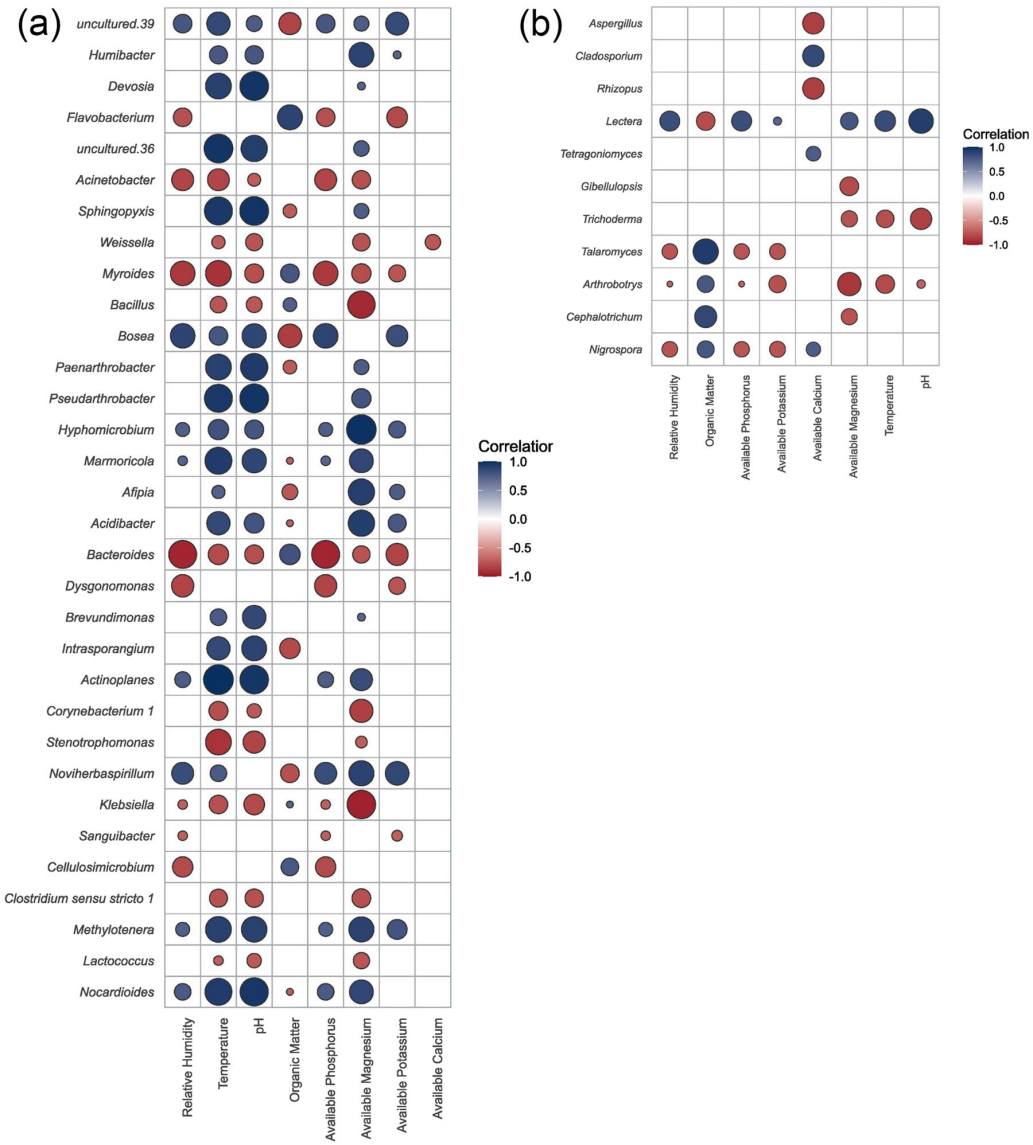
Pearson correlation of environmental factors and (a) bacterial and (b) fungal genera associated with potato tubers. Only statistically significant correlations are displayed (*p* < 0.05). Correlation coefficients are size‐based on statistical significance and colored according to a color scale ranging from dark red (negative correlations) to dark blue (positive correlations).

The bacterial community exhibited various kinds of responses to environmental factors. Several bacterial taxa, including *Paenarthrobacter*, *Sphingopyxis*, and *Methylotenera*, exhibited positive correlations with both pH and temperature, suggesting that these bacteria prefer slightly alkaline and warmer soils. These conditions were in samples from Tak province. In contrast, taxa such as *Flavobacterium*, *Acinetobacter*, and *Klebsiella* exhibited a negative correlation with available magnesium and phosphorus, indicating potential sensitivity to nutrient‐rich soils (Figure 6a). The fungal community, on the other hand, responded more strongly to organic matter and nutrient levels. Organic matter showed a positive correlation with genera such as *Talaromyces*, indicating a preference for high organic matter in the soil, as observed in Chiang Mai. *Lectera* showed a positive correlation with temperature, pH, and soil nutrients, but showed a negative correlation with organic matter. Several fungal taxa, such as *Nigrospora*, *Talaromyces*, and *Trichoderma*, showed negative relationships with magnesium and phosphorus, suggesting that high levels of nutrients might prevent them from surviving or functioning (Figure 6b). Overall, temperature and pH were found to be the primary factors influencing the composition of bacteria, although relative humidity, organic matter, and certain available nutrients (phosphorus, potassium, and calcium) had a lesser impact. In contrast, organic matter and soil nutrients have a greater effect on fungi than bacteria.

**Figure 7:**
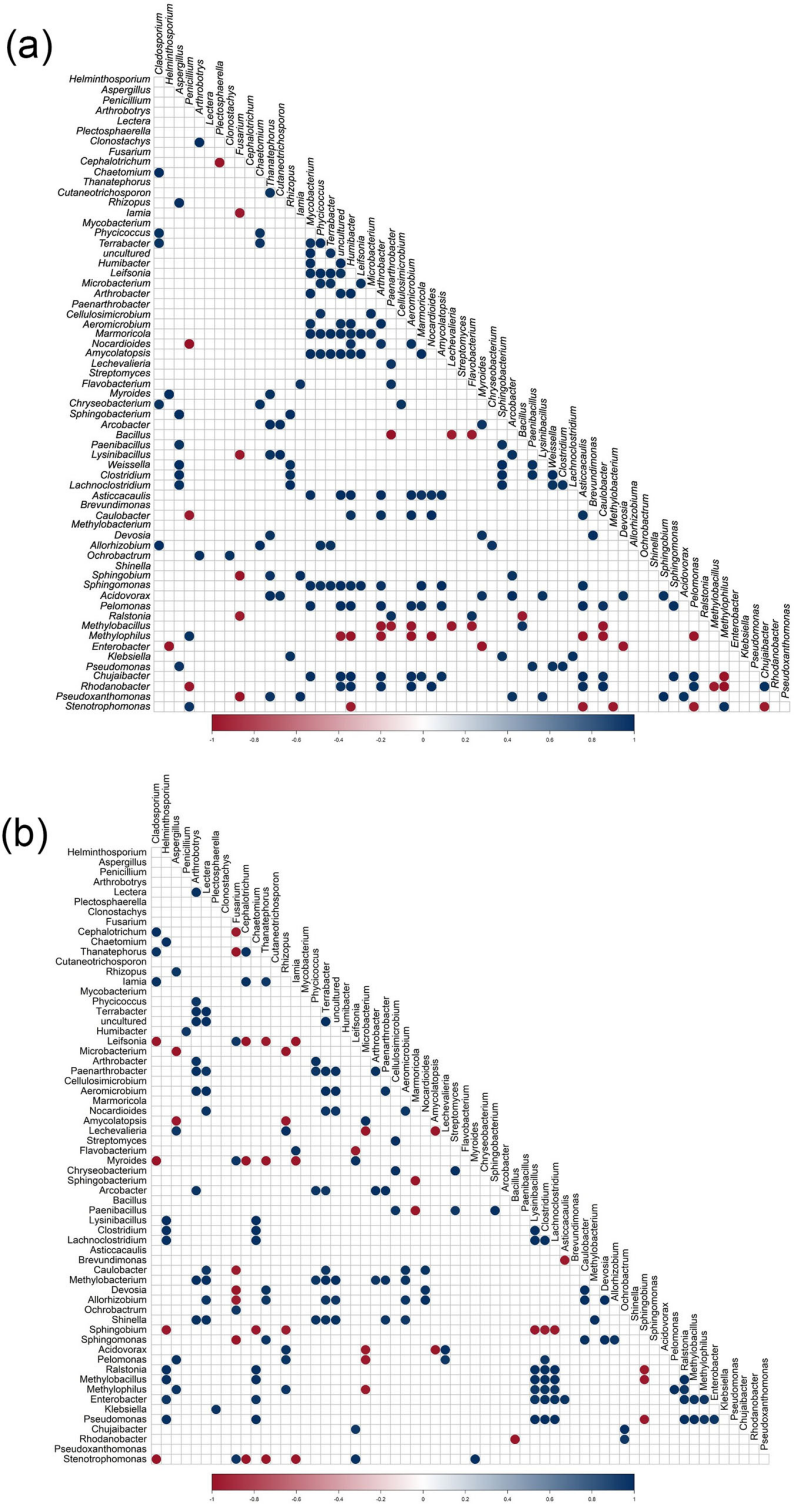
Pearson correlation analysis of the relative abundances of bacterial and fungal genera associated with potato tubers cultivated in the fields (a) Chiang Mai and (b) Tak. Only statistically significant correlations are displayed (*p* < 0.05). Correlation coefficients are colored based on a color scale ranging from dark red (negative correlations) to dark blue (positive correlations).

Microbial taxonomic correlation analysis showed clear patterns of interaction between the two groups: Tak and Chiang Mai. Bacterial intracorrelations (i.e., bacteria–bacteria correlations) reveal that the potato tubers grown in Chiang Mai had a more densely connected network of significant correlations, suggesting more tightly compacted microbial connections (Figure 7a). In contrast, the tubers grown in Tak had fewer significant connections, implying that microbial community dynamics were altered or modified (Figure 7b). For instance, *Enterobacter* showed strong negative correlations with other taxa in the microbial communities of the potato tubers cultivated in Chiang Mai; however, this trend shifted to strong positive correlations with other taxa in the tubers grown in Tak. *Streptomyces* also exhibited no significant relationships in the microbial communities of tubers grown in Chiang Mai, whereas it showed substantial associations in the potato tubers cultivated in Tak. Certain taxa, such as *Pseudomonas*, *Sphingobium*, and *Ralstonia*, also exhibited significant differences in their interaction profiles between the microbial communities of the potato tubers cultivated in Chiang Mai (Figure 7a) and Tak (Figure 7b), emphasizing the impact of environmental factors on microbial relationships.

Fungal‐intra correlation (fungi–fungi) analysis showed that the microbial communities of the potato tubers grown in Tak exhibited closer correlations than those grown in Chiang Mai. *Rhizopus* and *Aspergillus* showed significant positive associations in the microbial communities of the potato tubers grown under the different environmental conditions (Figure 7a,b). No significant correlations were observed in the fungal communities of the potato tubers grown in Chiang Mai; however, it is interesting that *Fusarium* exhibited negative relationships with *Thanatephorus* and *Cephalotrichum* in the tubers cultivated in Tak (Figure 7b).

The interkingdom (bacteria–fungi) correlation study indicated there were different microbial interaction patterns between the potato tubers grown in Tak and Chiang Mai. The positive relationships between bacteria and fungi were stronger for the potato tubers cultivated in Tak (Figure 7b) than in those grown in Chiang Mai (Figure 7a). For instance, *Helminthosporium* had a positive interaction with endospore‐forming bacteria (e.g., *Lysinibacillus*, *Clostridium*, and *Lachnoclostridium*) and *Enterobacter* (Figure 7b), while it exhibited a negative correlation with *Enterobacter* in potato tubers grown in Chiang Mai (Figure 7a); *Arthrobotrys* showed a positive relationship with actinobacteria (i.e., *Terrabacter*, *Arthrobacter*, and *Paenarthrobacter*), and *Chaetomium* exhibited a positive correlation with *Lysinibacillus*, *Clostridium*, *Lachnoclostridium*, *Ralstonia*, *Methylobacillus*, *Enterobacter*, and *Pseudomonas* (Figure 7b), while this correlation was negative for the tubers grown in Chiang Mai (Figure 7a). These findings indicate that the environmental factor tends to stabilize potato tuber microbial communities and promote more significant interkingdom microbial interactions.

### 3.7 Predicted Functional Profiles of the Potato Tuber Microbiome

**Figure 8:**
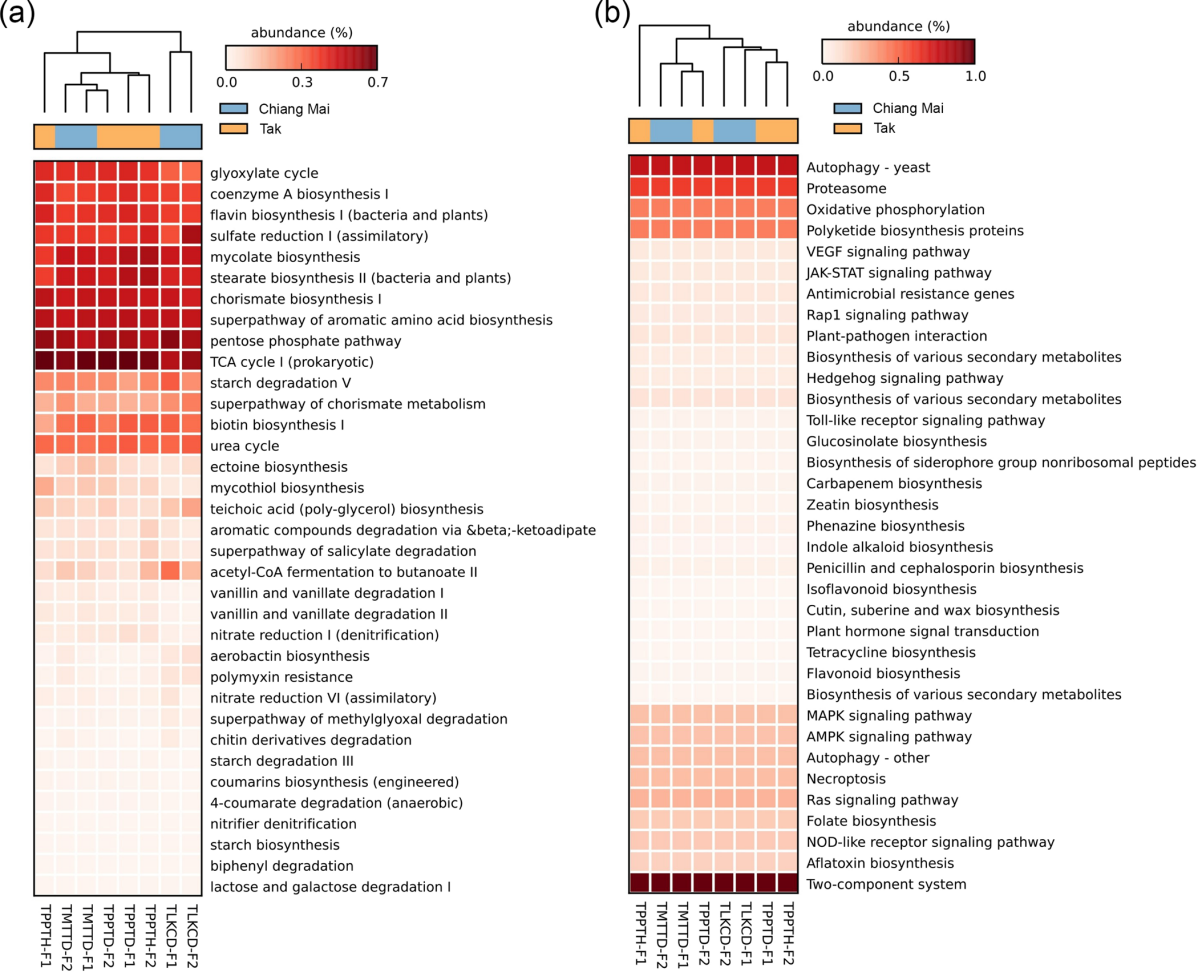
Heatmap of the top 35 predicted functions of the bacterial and fungal communities associated with potato tubers grown in Chiang Mai and Tak Provinces, analyzed through (a) PICRUSt2 for bacterial communities and (b) FunFun for fungal communities.

The bacterial functional profiles based on the MetaCyc database highlight various biological pathways, especially those related to anabolic and catabolic metabolism (Figure 8a). The most dominant (i.e., the 11 most abundant) metabolic pathways in the bacterial communities of the potato tubers grown in Chaing Mai and Tak are TCA (tricarboxylic acid) cycle I (prokaryotic), pentose phosphate pathway (PPP), the superpathway of aromatic amino acid biosynthesis, chorismate biosynthesis I, stearate biosynthesis II (bacteria and plants), mycolate biosynthesis, sulfate reduction I (assimilatory), flavin biosynthesis I (bacteria and plants), the glyoxylate cycle, and the urea cycle (Figure 8a). Although the top 11 predominant pathways were similar in proportion, some differences were observed, though they were not statistically significant (*p* > 0.05). For example, acetyl‐CoA fermentation to butanoate II was slightly more abundant in the bacterial communities of the potato tubers grown in Chiang Mai, and nitrate reduction VI (assimilatory) and polymyxin resistance were slightly less abundant in the microbiomes of the tubers cultivated in Tak (Figure 8a).

According to the Kyoto encyclopedia of genes and genomes (KEGG), the functional profiles of fungal organisms indicate their involvement in various metabolic processes, environmental information processing, and cellular processes. The top 10 pathways found in the fungal communities of the potato tubers grown in Chiang Mai and Tak include the two‐component system, autophagy‐yeast, proteasome, oxidative phosphorylation, polyketide biosynthesis protein, Ras signaling, MAPK signaling, AMPK signaling, autophagy‐other, and necroptosis pathways (Figure 8b).

## 4 Discussion

In this study, we demonstrated the significant influence of the soil environment on the diversity, taxonomic composition, functional potential, and correlation patterns of potato tuber‐associated microbiota. Exploring the keystone taxa and their correlations with the host plants, pathogens, and beneficial microbes as a foundation for microbiome‐based strategies to enhance crop health and productivity (Trivedi et al. 2020). Interestingly, the two main environmental factors influencing the composition of the microbial community were soil pH, organic matter. Although temperature has an effect, previous studies indicate that increased soil temperature alone may have minor impacts if changes in moisture or pH do not occur (Schindlbacher et al. 2011). In terms of soil type, both Chiang Mai (clay loam) and Tak (sandy clay loam) share similar properties, with soils that support good water retention and air flow. This similarity suggests that soil type is likely a minor factor influencing microbial community composition in this study, as it does not differ substantially between the two locations (Figure 3). Consistent with this, our results suggest that soil pH and organic matter exert a more pronounced influence on microbial community composition than temperature and soil type. This is particularly evident in the observed differences in the microbial composition of tubers from Tak and Chiang Mai, which we attribute to different strategies of soil management: Tak soils were supplemented with dolomite, which raised the pH levels of the soil, whereas Chiang Mai soils received manure, which raised the organic matter in the soil. These results highlight how important pH and organic matter regulation are in shaping plant‐associated microbiomes, particularly when conducted with soil amendments including dolomite or manure.

In this study, influence soil microbial several as potential beneficial bacteria, including *Microbacterium* and *Rhodanobacter*, were enriched in the microbial communities associated with the potato tubers grown in Tak (Figure 1a). Previous studies have revealed that members of *Microbacterium* (e.g., *M. foliorum* and *M. phyllosphaerae*) are dominant in neutral to alkaline rhizosphere soils. They also play crucial roles in plant growth promotion by increasing chlorophyll content and producing indole‐3‐acetic acid (IAA) (Yu et al. 2024) and in potato pathogen suppression by decreasing the incidence of potato bacterial wilt caused by *Ralstonia solanacearum* (Bahmani et al. 2021). Our results support these findings, showing that *Ralstonia* was less abundant in potato tubers exhibiting a high abundance of *Microbacterium* (TPPTD‐F1, TPPTD‐F2, and TPPTH‐F1) (Figure 1b). Members of *Rhodanobacter*, nitrogen‐fixing bacteria, are typically found in mildly acidic to neutral potato rhizosphere soils (Bak et al. 2025) and have been identified as endophytes in sweet potato tubers (Khan and Doty 2009). This genus can produce IAA, which promotes root growth in the host plant, thereby increasing the uptake of nitrogen from the soil (Khan and Doty 2009).

Nevertheless, our results indicate that *Rhodanobacter* was found to be associated with the potato tubers grown in Chiang Mai (TMMTTD‐F1 and TMMTTD‐F2) in a proportion similar to that of those grown in Tak (TPPTH‐F1 and TPPTH‐F2) (Figure 1b). This was not surprising, as members of *Rhodanobacter*, such as *R. lycopersici*, (Woo et al. 2024), and *R. humi* (Dahal and Kim 2017) are acid‐ and alkali‐tolerant bacteria capable of surviving at pHs ranging from 3.0 to 13.0. Interestingly, *Weissella*, a lactic acid bacterium, was absent in the potato tubers cultivated in Tak (the fields treated with dolomite) (Figure 1d). This phenomenon is consistent with the findings of a previous study that indicated lime (CaO) or its derivatives are effective in inhibiting lactic acid bacteria growth (Lee et al. 2020).

As shown in Figure 1b, *Streptomyces*, *Bacillus*, *Microbacterium*, *Nocardioides*, and *Devosia* were the most abundant bacterial genera in all the potato tuber microbial communities sampled. This result is consistent with the findings presented by Buchholz in a previous study (Buchholz et al. 2019), which reported that these bacterial genera were most abundant in potato seed tubers. Furthermore, the other abundant genera found in our study, such as *Ralstonia*, *Enterobacter*, *Allorhizobium*‐*Neorhizobium*‐*Pararhizobium*‐*Rhizobium*, *Rhodanobacter*, and *Pseudomonas*, were similar to the dominant genera associated with the tare soil microbiome of seed potatoes (Delventhal et al. 2023). These findings suggest that bacteria associated with potato tubers could be recruited from the soil, especially the geocaulosphere.

In the fungal communities, the genus *Fusarium* exhibited a high abundance in all the sampled potato tubers (Figure 1d). Although it has been reported that treating soil with lime, which raises the pH of soil, reduced the incidence of Fusarium wilt in various plants, such as spinach (Gatch and du Toit 2017), and banana plant (Segura et al. 2022), Gordon et al. (2019) reported that *Fusarium* species (e.g., *F. oxysporum* f. sp. *fragariae*) often grow faster in natural acidic soil (pH 5.0) and neutral soil (pH 7.0). Their study also highlighted that the observed reduction in Fusarium wilt incidence at higher pH levels was influenced by the greater activity of microbial communities competing with *Fusarium* in planta colonization. Moreover, our results are consistent with an earlier study conducted by Akosah et al. (2021), that indicated that *Fusarium* species were dominant during the flowering stage of potato and could persist in acidic to neutral soils until the senescence stage. Our findings also illustrate that *Plectosphaerella* was the most abundant genus and represented a core fungal genus of potato tubers (Figure 2) grown in both Chiang Mai and Tak. These results are consistent with the findings of a previous study in which *Plectosphaerella* was identified as a member of the potato tare soil microbiome (Delventhal et al. 2023). A previous study reported that *P. cucumerina* strain GJD‐8 caused potato wilt disease in Mongolia, China (Gao et al. 2016); however, *P. cucumerina* strain 380408 could reduce the potato cyst nematode population by up to 60% in the field (Atkins et al. 2003). These findings suggest that the presence of *Plectosphaerella* species may play a role in both disease dynamics and pest suppression.

Although previous studies have reported that members of *Chaetomium*, such as *C. globosum* and *C. lucknowense*, grow favorably and can survive in acidic to alkaline environments (Fogle et al. 2008; Hung et al. 2015), the growth of *Chaetomium* species often depends on soil organic matter and available phosphorus (Prokhorov and Linnik 2011; Ma et al. 2023). High levels of organic matter and available phosphorus in the soil could enhance the growth rate and biomass of *Chaetomium*. These studies support our findings of *Chaetomium* being highly abundant in the potato tubers grown in the field (TMTTD‐F2) with high a organic matter content (39,300 mg·kg**^−^** ^1^) and the field (TPPTH‐F2) with a high available phosphorous content (93 mg·kg**^−^** ^1^) (Figure 1d). We also found that *Arthrobotrys* species were less abundant in the potato tubers grown in Tak (with a pH close to a neutral) than in Chiang Mai (with a pH close to a that of a strong acid) (Figure 1d). These results agree with previous reports that nematode‐trapping fungi, such as *Arthrobotrys oligospora*, prefer an acidic soil environment (pH 4.4–5.1) (Farrell et al. 2006); however, some species (e.g., *A. haptophyla* and *A. conoides*) favor a neutral soil. Moreover, a high abundance of the nematophagous fungus *Arthrobotrys* in soil could indicate the presence of root‐knot (*Meloidogyne* sp.) and root‐lesion (*Pratylenchus* sp.) nematodes in the soil (Jaffee 2000).

Our analysis indicated that the alpha diversities of the microbial communities associated with all the potato tubers sampled did not differ (Figure 4a,b). However, there were differences in beta diversity, which were influenced by the soil amendments used (Figure 4c,d). Applying soil with manure can raise the organic matter, and dolomite can raise the pH of soil from acid to neutral or alkaline, constituting the main factor altering soil microbial structure (Qi et al. 2018; Wang et al. 2024). Moreover, it has been reported that high organic matter content can enhance the growth of functional bacteria and fungi (Coonan et al. 2020), while pH conditions also shape microbial communities, with neutral to slightly alkaline environments favoring bacterial growth, and acidic conditions promoting fungal dominance (Rousk et al. 2009).

As shown in Figure 5a, *Afipia* was the genus whose abundance increased most significantly in the potato tubers grown in Tak. This finding agrees with earlier reports that several *Afipia* species (e.g., *A. felis*, *A. broomeae*, and *A. clevelandensis*) grow well within a broad range between pH 6.0 and 7.5 but poorly in soils with a pH lower than 6.5 (Müller. 1995). The abundance of *Bacteroides* and *Klebsiella* was slightly lower in the potato tubers grown in Tak; however, their abundance increased in the potato tubers grown in Chiang Mai, which had a pH close to acidic conditions and high organic matter (Table 2). These genera are well known for their tolerance of and adaptability to various environments (Kontro et al. 2005; Nojoumi et al. 1995). Regarding fungal differential composition, interestingly, the abundance of *Trichoderma*, a well‐known broad‐spectrum biocontrol agent, decreased in the potato tubers cultivated in the fields treated with dolomite (Tak) (Figure 5b). This result aligns with the findings of a previous study in which applying dolomite to soil was reported to reduce the growth rate of *Trichoderma* species, such as *T. koningiopsis* and *T. virens* (Botero et al. 2015). The abundance of most fungal species in the genera *Lectera*, *Pyrenochaetopsis*, *Stachybotrys*, *Lasiodiplodia*, *Periconi*, and *Cephalosporium* was found to be greater in the potato tubers grown in Tak (Figure 5b), but there are no direct reports of these fungi surviving in neutral soil environments. Most fungi can survive in a stressful environment by adapting or producing resistance structures such as sclerotia or sexual spores (ascospores, basidiospores, zygospores, and oospores) (Branco et al. 2022). These adaptations enable fungi to tolerate various environments.

Cooperative and competitive interaction among microbial species can alter community stability (Dassen et al. 2017; Gao et al. 2021). In this study, microbes associated with potato tubers from Tak exhibited altered and less dense networks (Figure 7b) compared to those from Chiang Mai (Figure 7a), indicating changes in the dynamics of the microbial population. Several taxa, such as *Enterobacter*, changed their interactions with the fungus *Helminthosporium* from negative to positive in the tubers grown in Tak. Meanwhile, *Streptomyces* exhibited strong, consistent interactions with other microbes in the tubers from both fields. These findings align with previous research, highlighting that soil amendments can enhance microbial interactions and foster positive associations among soil microorganisms (Ma et al. 2021). Likewise, fungal relationships were more robust in the fields subjected to dolomite amendment, and bacterial relationships were more robust in high organic matter, suggesting improved community connectivity and stability. For instance, increased antagonistic interactions between *Fusarium* and *Thanatephorus* or *Cephalotrichum* indicate a complex interaction that could be affected by dolomite (Figure 7b), and *Humibacter* are increased interactions that are affected by manure (Figure 7b), potentially altering competitive relationships in potato tuber fungal communities. According to the interkingdom analysis, Tak facilitated the development of more extensive microbial networks within both the bacterial and fungal communities of the potato tubers (Figure 7b). This observation proves the findings from a previous study, which illustrated how the introduction of microbial entities can enhance functional diversity and modify existing networks (Deng et al. 2019). The increasing complexity of these networks is intended to improve ecosystem function and stability, as more intricate microbial interactions contribute to enhanced nutrient cycling and plant health (Tao et al. 2018). Our findings demonstrate that the soil amendment can impact microbial populations, providing valuable insights for enhancing soil management strategies to improve agricultural productivity and ecosystem health. Understanding these dynamics can aid in developing strategies that support environmentally sustainable farming practices by integrating beneficial microbial interactions.

Focusing on shifts in the microbial community, potatoes are commonly cultivated after rice in the lowland areas of northern Thailand (Kittipadakul et al. 2016). Several previous studies reported that *Pseudomonas*, *Curtobacterium*, *Methylobacterium*, *Sphingomonas*, *Microbacterium*, *Flavobacterium*, and *Chryseobacterium* are the core bacterial taxa of rice rhizosphere and bulk soils (Zhang et al. 2022; Luo et al. 2025), whereas *Trichoderma*, *Aspergillus*, *Neurospora*, *Staphylotrichum*, and *Thanatephorus* are the predominant fungal taxa (Zhou et al. 2023; Zhu et al. 2024). In comparison, our results highlighted that the core microbial taxa associated with potato tubers grown in the two main potato production areas of the lowland regions, Chiang Mai and Tak Provinces, comprised *Streptomyces*, *Bacillus*, *Fusarium*, and *Plectosphaerella* (Figure 2). This observation aligns with the study by Zhang et al. (2025), which indicates that the genus *Streptomyces* typically increases during the cultivation of potatoes. Moreover, our findings support the study by Zong et al. (2024), which has illustrated that crop rotation can directly alter the diversity and composition of soil microbial communities.

In terms of plant growth and environmental conditions, Chiang Mai exhibited a higher potato yield than Tak, likely due to its higher organic matter content, which creates a favorable environment for potato growth, along with acidic soil pH (4.6–4.9), a range commonly associated with productive potato fields (Chen et al. 2022). In contrast, although Tak soils had lower organic matter, which may limit plant productivity, they provided a more suitable environment for beneficial microorganisms, such as *Azohydromonas*, well known for its nitrogen‐fixing ability and role in promoting plant growth (Dahal et al. 2021). These findings suggest that while Chiang Mai's soil chemistry directly supports plant productivity, Tak's microbial environment may offer long‐term benefits through microbial‐mediated nutrient cycling, highlighting the need to balance both soil chemistry and microbiome management to optimize potato yield.

Regarding the relationship between soil properties and potato production yield, as shown in Table 1, potatoes cultivated in acidic clay loam soil (pH 4.6–4.9) in Chiang Mai Province yielded more per hectare than those cultivated in neutral sandy clay loam soil (pH 6.6–6.8) in Tak Province (Table 2). The result is consistent with the study by Popescu (2020), which illustrated that potatoes generally perform best in acidic soil (pH 4.8–5.5). Our finding also agrees with several previous reports, which demonstrate that loam and clay loam soils are more suitable for potato cultivation due to their high organic matter content and rich microbial communities, thereby promoting high potato tuber yields (Koco et al. 2020; Tian et al. 2023; Carruthers and Congreves 2025). The slightly lower potato yield in Tak Province compared to Chiang Mai (Table 1) can be attributed to the application of lime or dolomite. Dolomite application helps regulate soil pH, reduce bacterial wilt pathogen, and favor beneficial bacteria; however, overuse of dolomite could reduce soil organic matter and microbial activity (Bergtold and Sailus 2020). Based on our findings, clay loam soil with a pH ranging from 4.6 to 4.9 is suitable for potato cultivation. Furthermore, dolomite application should be considered an optional practice in areas with a history of bacterial wilt disease to manage the disease under acidic soil conditions.

Interestingly, there are no significant differences in the predicted functional profiles of the bacterial and fungal communities associated with the potato tubers grown in the dolomite‐treated or untreated fields. Our findings indicate that microbial functional activities in potato tubers are stable and resilient to shifting environmental factors or changes in community composition. The most common functions in the bacterial communities of potato tubers were associated with TCA (tricarboxylic acid) cycle I (prokaryotic), the PPP, the glyoxylate cycle, and chorismate biosynthesis I (Figure 8a). The TCA cycle, PPP, and glyoxylate cycle served as fundamental metabolic reactions for ATP generation (Caspi et al. 2018). Additionally, we found that the chorismate biosynthesis I pathway plays an essential role in synthesizing secondary metabolites and aromatic amino acids that support plant defense mechanisms and health. For example, *B. subtilis* and *Escherichia coli* use this pathway to synthesize intermediates involved in producing secondary metabolites (Caspi et al. 2014; Zhong et al. 2020).

The fungal communities in potato tubers also revealed important mechanisms that enable fungi to adapt to and survive environmental stress (Figure 8b). For instance, the production of lipids, such as wax, cutin, and suberin, is essential for protecting cell integrity and defending against environmental stressors. Furthermore, these fungi possess signaling pathways related to cell survival, such as the MAPK (mitogen‐activated protein kinase) signaling pathway, which responds to various stressors, including UV light and temperature changes; assists in virulence; enables cell‐to‐cell communication; influences interactions between fungi and plants; and responds to damage‐associated molecular patterns (DAMPs), such as in *Fusarium* (González‐Rubio et al. 2019; Martínez‐Soto and Ruiz‐Herrera 2017). An interesting feature of the fungal communities in the potato tubers was the presence of the AMPK (AMP‐activated protein kinase) signaling pathway (Figure 8b). AMPK, a ubiquitous sensor of cellular energy and nutritional status in eukaryotic cells, is responsible for controlling the carbon source utilization, cell proliferation, spore formation, pathogenicity, and stress response in both beneficial and phytopathogenic filamentous fungi, including *A. oligospora*, *Fusarium graminearum*, and *Magnaporthe oryzae* (Wang et al. 2022). Our results highlight that the soil amendment did not significantly affect the microbial functions of the microbial communities associated with potato tubers, and most functions were related to energy metabolism, cellular processes, and environmental adaptations.

## 5 Conclusion

In this study, we investigated how soil factors affect microbial diversity, composition, interactions, and functions associated with potato tubers cultivated in the lowland areas of northern Thailand. The dominant bacterial phyla were *Proteobacteria*, *Actinobacteria*, and *Firmicutes*, while fungal communities were dominated by the phylum *Ascomycota*. The core taxa of the potato tubers included *Streptomyces*, *Bacillus*, *Fusarium*, and *Plectosphaerella*. Although alpha diversity did not differ significantly between Tak (dolomite‐treated) and Chiang Mai (manure‐amended) soils, beta diversity analysis revealed significant shifts in the microbial community. The novelty of our findings lies in providing direct evidence that specific soil physicochemical properties, particularly pH and organic matter content, serve as the principal determinants of the potato tuber‐associated microbiome structure, effectively influencing the balance between potential plant pathogens and beneficial taxa. The primary contribution of these results to potato cultivation is the provision of an evidence‐based framework for sustainable disease and quality management. Specifically, by demonstrating that the tuber microbiome is an inducible trait, the present study strongly advocates for the strategic modification of soil properties as a potent, nonchemical leverage point to actively enhance tuber health, suppress localized pathogen populations, and ultimately optimize potato yield and quality in this economically vital agricultural region.

## Author Contributions

**Pipat Macharoen:** methodology, software, formal analysis, investigation, data curation, writing – original draft preparation, writing – review and editing, visualization, funding acquisition. **Wuttichai Mhuantong:** conceptualization, software, validation, formal analysis, writing – original draft preparation, writing – review and editing, visualization. **Thippawan Wannawong:** formal analysis, writing – review and editing. **Wiphawee Leesutthiphonchai:** methodology, resources, writing – review and editing. **Onuma Piasai:** resources, writing – review and editing. **Somboon Tanasupawat:** writing – review and editing. **Nakarin Suwannarach:** conceptualization, methodology, validation, investigation, resources, writing – original draft preparation, writing – review and editing. **Nattakorn Kuncharoen:** conceptualization, methodology, validation, formal analysis, investigation, resources, writing – original draft preparation, writing – review and editing, supervision, project administration, funding acquisition. All authors have read and agreed to the published version of this manuscript.

## Ethics Statement

The authors have nothing to report.

## Consent

The authors have nothing to report.

## Conflicts of Interest

The authors declare no conflicts of interest.

## Data Availability

The raw sequencing data have been deposited in the NCBI Sequence Read Archive (SRA) database under the accession numbers PRJNA1235626 (16S) and PRJNA1235648 (ITS). The datasets analyzed in the current study are available from the corresponding author upon reasonable request.
